# Protein-Modulated Stimuli-Responsive Hydrogels Based on Methacrylated Bovine Serum Albumin and pNIPAm: pH- and Temperature-Dependent Drug Release Behavior

**DOI:** 10.3390/gels12030263

**Published:** 2026-03-22

**Authors:** Muge Sennaroglu Bostan

**Affiliations:** Department of Chemical Engineering, Engineering Faculty, Marmara University, Aydınevler, Maltepe, Istanbul 34854, Türkiye; msennaroglu@marmara.edu.tr

**Keywords:** BSA, NIPA, GMA, dual responsive hydrogels, protein based hydrogels, 5-fluorouracil (5-FU), gastrointestinal drug release

## Abstract

Hydrogels are widely investigated as drug carriers for cancer therapy due to their ability to provide sustained release and reduce systemic side effects. In this study, MeBSA–PNIPAm hydrogels were developed as dual-temperature and pH-responsive systems for gastrointestinal delivery of 5-FU. MeBSA was successfully synthesized using glycidyl methacrylate and confirmed by FTIR and ^1^H-NMR analyses. Hydrogels with varying MeBSA/NIPA ratios were prepared via redox polymerization. DSC results showed that increasing MeBSA content shifted the phase transition temperature of hydrogels, while TGA analysis revealed enhanced thermal stability with higher MeBSA incorporation. Temperature-dependent swelling experiments further demonstrated that the VPTT slightly shifted depending on the surrounding pH, indicating that the thermoresponsive behavior of the hybrid network is influenced by the pH-dependent charge state of the protein component. Swelling studies performed at 30, 37, and 40 °C and at pH 1.2 and 7.4 confirmed dual-responsive behavior. Drug loading efficiencies above 70% were achieved for all formulations. In vitro release studies at 37 °C demonstrated distinct composition-dependent release profiles. During the first 2 h, all hydrogels exhibited controlled and limited release without burst behavior under acidic conditions. Following the transition to pH 7.4, a composition-dependent increase in drug release was observed. GEL 4 achieved the fastest and highest cumulative release (91%), whereas GEL 1 provided the most sustained release over 72 h (32%). Kinetic analysis indicated diffusion-controlled release, best described by the Weibull and Korsmeyer–Peppas models. Cytocompatibility tests showed that fibroblast viability improved with increasing MeBSA content. Overall, protein-modulated dual-responsive hydrogels offer tunable and biocompatible platforms for stimuli-responsive gastrointestinal drug delivery applications.

## 1. Introduction

Soft hydrogel systems have gained great attention in drug delivery applications due to their three-dimensional, hydrated and swollen network structure, with additional superior features that resemble biological matrices. Unlike other solid polymeric materials, hydrogels allow water-mediated transport while maintaining network integrity through physical or chemical crosslinking, enabling simultaneous drug loading and controlled diffusion. The balance found between mechanical stability and molecular mobility makes hydrogels suitable for various biomedical applications, where interaction with aqueous biological environments and living tissues are needed [1,2,3,4].

Beyond synthetic polymer networks, attention has been directed toward protein-based natural-origin hydrogels, which enhance biological integration and provide functional advantages in material design. Proteins have folded structures, functional parts, and various intermolecular interaction sites that are absent in conventional synthetic polymers [1]. More broadly, hybrid protein–polymer materials have attracted increasing interest, as they combine the biological functionality of proteins with the structural tunability of synthetic polymers. Various applications have been reported in the literature, including protein–polymer conjugates, polymer-modified protein networks, and hybrid biomaterials designed for biomedical applications such as drug delivery, tissue engineering, and biosensing [5,6]. In addition, surface-grafted polymer brush architectures have been proposed to control protein immobilization and biointeractions at polymer interfaces [7]. Although these systems are mainly designed for biointerface engineering, they demonstrate the versatility of integrating proteins into synthetic polymer frameworks. Compared with surface-confined architectures, incorporating proteins directly into bulk hydrogel networks represents an alternative strategy to generate stimuli-responsive biomaterials capable of regulating swelling behavior and drug transport in aqueous environments.

As a result, protein-based hydrogels often display enhanced biocompatibility, biodegradability, and responsiveness, while offering additional drug matrix interaction opportunities through hydrophobic parts, electrostatic domains, and hydrogen-bonding regions [2,3]. In the literature, a wide range of protein-based hydrogels has been reported, originating from different protein structures and architectures. Fibrillar proteins such as collagen [4], gelatin [8], and silk fibroin [9], as well as globular proteins like serum albumin [10], lysozyme, and β-lactoglobulin are some of the examples. Thanks to their intrinsic biocompatibility and structural adaptability, such hydrogels have been widely investigated for drug delivery and related biomedical applications [3]. Among these, BSA is a particularly attractive globular protein for hydrogel design and applications.

BSA is a non-glycosylated protein with high aqueous solubility and structural stability and a well-known safety profile [2]. Its structural similarity to human serum albumin (HSA), commercial availability and low cost has led to its frequent use in biomedical and pharmaceutical research. BSA contains various hydrophobic binding parts and charged domains, which allow reversible interactions with various small drug molecules. BSA has a molecular weight of nearly 66 kDa and is composed of more than 580 amino acid residues [11]. Moreover, its chemical structure includes multiple disulfide bonds, the free sulfhydryl (–SH) group and diverse functional groups such as amino (–NH_2_) and carboxyl (–COOH). These functional groups enable various chemical modifications and further functionalization of BSA, allowing it to play an active role in hydrogel systems [12]. Moreover, albumin not only provides mechanical support but also can directly participate in drug retention and release via these functional groups, which promote protein–drug interactions [13].

BSA exhibits pH-dependent charge regulation, with an isoelectric point in the range of pH 4.7–4.9. The net surface charge is zero at this pH, which causes reduced electostatic repulsion and increases the tendency of BSA molecules to aggregate and adopt a more compact structure. With increasing pH, the surface charge of BSA becomes increasingly negative and enhances electrostatic repulsion, stabilizing BSA molecules and promoting a more expanded conformation [14]. As a result, BSA-based hydrogels are functional carriers capable of modulating drug availability in response to environmental conditions [15]. These conformational and charge variations facilitate protein–drug interactions [16]. Although BSA is not known as a pH-responsive polymer, these pH-dependent variations can significantly influence swelling and molecular transport when BSA is incorporated into hydrogel networks. This behavior is especially relevant for environments such as the gastrointestinal tract and tumor tissues, where pH gradients are present [17,18].

BSA-based hydrogels can be formed through pH, heat and ethanol-induced physical methods non-covalently; however, the resulting network structure strongly depends on factors such as concentration, pH, temperature, and time, and it is not yet fully understood [12,19]. Exposing to high temperature may cause partial denaturation of BSA, damage to the structure and reductio of its bioactivity. On the other hand, several studies have shown that hydrogels prepared under mild physical conditions have lower mechanical strength than those prepared under acidic conditions or at high temperatures [15].

Albumin hydrogels can also be prepared via covalent crosslinking, which provides improved mechanical strength compared to physically crosslinked systems. Unlike physically prepared BSA systems, chemically crosslinked BSA hydrogels exhibit superior structural stability and mechanical integrity compared to physically prepared ones [12]. The fabrication of chemically cross-linked BSA hydrogels mostly includes the addition of crosslinkers such as glutaraldehyde, epichlorohydrin, and tetra(hydroxymethyl)phosphonium sulfate, which raises toxicity concerns, remaining a critical limitation in biomedical applications [12].

BSA also has been extensively modified through its reactive functional groups to introduce additional reactive parts into the backbone to enable further conjugation and crosslinking reactions. Modifying lysine residues using N-hydroxysuccinimide (NHS) ester chemistry [20], targeting the single free thiol group via the maleimide-thiol coupling method [21], and grafting polymerizable groups onto the protein backbone [22] are some representative examples of these approaches. Methacrylation of BSA using glycidyl methacrylate (GMA) [23] is a particularly attractive approach to introduce polymerizable vinyl groups, allowing BSA to directly participate in the covalent hydrogel network structure. This method enables the fabrication of hydrogels without the use of additional crosslinkers, preserving the protein structure while reducing potential toxicity. There are several successful examples of the methacrylation of BSA reported in the literature for the fabrication of protein-based hydrogels. Ozkahraman et al. reported photocrosslinkable methacrylated BSA-based conductive hydrogel patches reinforced with reduced graphene oxide for potential cardiac applications, which demonstrated tunable mechanical properties, slower degradation, mineralization capability, and high cell viability [24]. In addition, GMA-modified BSA systems have been reported as 3D hydrogels obtained via the two-photon polymerization method, preserving biocompatibility and pH responsiveness [23]. Moreover an albumin methacryloyl-based cryogel platform mimicking liver microarchitecture was developed, providing a highly porous 3D system that enhanced liver-specific functionality and enabled effective detection of drug-induced hepatotoxicity [25]. These studies confirm that methacrylated BSA provides a versatile and biologically favorable platform for biomedical applications with various purposes.

Temperature-responsive polymers offer direct control over the swelling–deswelling properties of networks. PNIPAm is a well-known and often-studied thermoresponsive hydrogel exhibiting a lower critical solution temperature (LCST) around 32 °C. In aqueous solution, LCST corresponds to a reversible coil–globule transition of linear PNIPAm chains, while in chemically crosslinked hydrogel networks, this thermally induced transition is more appropriately defined as the volume phase transition temperature (VPTT), reflecting dehydration-mediated structural rearrangement within a covalently crosslinked network [26]. Molecular-level analyses have revealed that the LCST behavior of PNIPAm originates from a competitive balance between polymer water hydrogen bonding and hydrophobic interactions within the isopropyl groups. From a thermodynamic perspective, this can be explained using the Gibbs free energy of mixing (ΔGₘ = ΔHₘ − TΔSₘ). The transition temperature results from the balance between polymer water hydrogen bonding and hydrophobic dehydration of the PNIPAm chains. Below LCST, the favorable hydrogen bonding between amide groups and water contributes to the negative enthalpy of mixing, whereas dehydration of the hydration shell and enhanced segment–segment hydrophobic interactions drive the coil-to-globule transition above the LCST. The critical solution temperature (CST) can therefore be described thermodynamically as CST = ΔHₘ/ΔSₘ, where variations in enthalpic and entropic contributions determine the transition temperature [27]. In crosslinked and compositionally complex hydrogel systems, this transition typically occurs over a temperature range rather than as an abrupt collapse at a single temperature and may be influenced by network architecture and intermolecular interactions [28]. Thanks to this reversible dehydration–rehydration mechanism, PNIPAm-based hydrogels have been widely employed to regulate temperature-triggered swelling behavior and drug release in controlled drug delivery systems [29,30]. However, these hydrogels have limited mechanical strength and poor biodegradability when used alone. In recent years, combining PNIPAm with natural biomolecules has emerged as an effective way to overcome these limitations and to improve the biological performance of thermoresponsive hydrogels.

In our previous studies, we successfully developed PNIPAm–biopolymer hybrid hydrogels with controllable swelling and improved stability. These systems showed promising results for controlled and targeted drug delivery applications [29,31]. In this context, combining methacrylated BSA with PNIPAm offers a promising strategy to develop hybrid stimuli-responsive hydrogels.

5-FU is an effective chemotherapeutic agent for colorectal [32], gastric [33], pancreatic [34], breast, head and neck, and skin cancers [35]. Despite this, it has some pharmacokinetic limitations, such as uncontrolled peak plasma concentrations, rapid metabolism, extremely short plasma half-life, systemic toxicity and severe side effects. Previous studies showed that the antitumor efficacy of 5-FU mainly depends on exposure duration rather than on plasma concentration levels. Due to its small size and high water solubility, 5-FU is difficult to retain in conventional carriers and is often released too quickly. Protein-based hydrogels, especially those containing albumin, offer advantages by combining physical entrapment with protein–drug interactions. When integrated into temperature-responsive networks, these interactions can be further controlled by environmental conditions. Therefore, maintaining sustained drug levels through continuous delivery may improve therapeutic outcomes and highlight the need for advanced controlled drug release systems [36].

In MeBSA–PNIPAm systems, PNIPAm may provide temperature-triggered volume changes, while MeBSA could contribute biocompatibility, pH-dependent behavior, and potential drug-binding interactions. The covalent incorporation of MeBSA into the network may enable hydrogel formation without external crosslinkers while preserving protein-mediated interactions within the matrix. This dual contribution could allow modulation of swelling and drug release behavior through compositional adjustment. Building on this concept, the present study incorporates methacrylated BSA into a PNIPAm-based network to develop dual pH- and temperature-responsive hydrogels for controlled 5-FU delivery.

In this study, methacrylated bovine serum albumin was copolymerized with N-isopropylacrylamide to form dual-responsive MeBSA–PNIPAm hydrogels without the use of additional crosslinking agents. Four formulations (GEL 1 to GEL 4) were prepared with increasing MeBSA content (15%, 20%, 25%, and 40%, respectively), corresponding to decreasing NIPA fractions (85%, 80%, 75%, and 60%), to systematically investigate the effect of composition on hydrogel performance. Therefore, the effect of increasing MeBSA content on thermoresponsive behavior, swelling, and drug loading can be systematically evaluated. The swelling behavior of the hydrogels was investigated at different temperatures, and the loading and release of 5-fluorouracil were examined under pH switching and physiological temperature conditions. Release data were analyzed using kinetic models to better understand the release mechanisms. Overall, this work presents a protein-based, crosslinker-free, and thermo- and pH-responsive hydrogel system with potential for controlled 5-FU delivery for intestinal route cancer treatment.

## 2. Results and Discussion

### 2.1. Structural Characterization of Methacrylated BSA (MeBSA)

**Figure 1:**
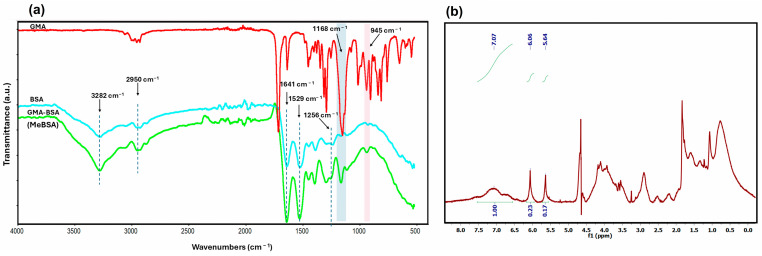
(**a**) FTIR spectra of GMA, BSA, and MeBSA; (**b**) ^1^H-NMR spectrum of MeBSA.

The FTIR spectra of GMA, BSA, and methacrylated BSA (MeBSA) are shown in Figure 1a. BSA displayed characteristic amide I (1641 cm^−1^), amide II (1529 cm^−1^), and amide III (1256 cm^−1^) bands, together with a broad absorption around 3282 cm^−1^, attributed to O–H and N–H stretching vibrations [37]. After modification with GMA, additional peaks appeared at 1168 and 945 cm^−1^, corresponding to –CH_2_ wagging and vinyl group vibrations of the methacrylate moiety. This modification occurs through an epoxide ring-opening reaction (aminolysis), in which the epoxide group of glycidyl methacrylate (GMA) reacts with the primary amine groups of lysine residues in BSA, leading to covalent attachment of methacrylate functionalities to the protein backbone [24]. The persistence of the main amide bands suggests that the overall protein backbone remained largely intact after modification. These spectral changes are in good agreement with previous reports on the methacrylation of albumin and other protein-based systems [23,24], where the appearance of vinyl-related peaks together with preserved amide bands has been interpreted as evidence of successful functionalization while indicating that the primary protein structure remains largely intact [23,38]. Such consistency with earlier studies further validates the successful grafting of GMA onto BSA in this work. To obtain a more direct molecular-level insight into the modification and to quantify the extent of grafting, ^1^H-NMR spectroscopy was subsequently employed.

Methacrylated BSA (MeBSA) was successfully synthesized via the reaction of BSA with GMA under controlled conditions to introduce polymerizable methacrylate functionalities onto the protein backbone. The reaction was carried out for 7 days to ensure a sufficient degree of methacrylation. Following the reaction with GMA, distinct vinyl proton resonances were detected at δ 6.06 and 5.64 ppm, accompanied by the GMA methyl peak at δ 1.84 ppm; as shown in Figure 1b, the ^1^H-NMR spectrum of MeBSA. In parallel, the resonance region around δ 2.9 ppm, attributed to lysine methylene protons in albumin, was still present after the reaction, indicating the existence of unreacted lysine side chains within the modified protein. This observation is consistent with the partial methacrylation of BSA, as only a fraction of available lysine residues is expected to participate in the reaction. The percent degree of methacrylation was determined by integrating the vinyl proton signals (δ 5.64 and 6.06 ppm) against the aromatic region (δ 6.27–7.43 ppm) according to Equation (1). The substitution level of GMA was calculated to be approximately 40% based on Equation (1).

(1) $$\%\;Degree\;of\;methacrylation=\frac{A_{\delta 5.64}+A_{\delta 6.06}}{A_{\delta 7.07}}\times 100$$

These findings indicate that the modification occurs through epoxide ring opening of the GMA and subsequent nucleophilic attack by lysine residues, in agreement with methacrylation pathways reported in the literature for BSA, while the successful incorporation of methacrylate functionalities was confirmed by FT-IR and ^1^H-NMR analyses [23,24,39,40].

### 2.2. Thermal Characterization of Hydrogels

**Figure 2:**
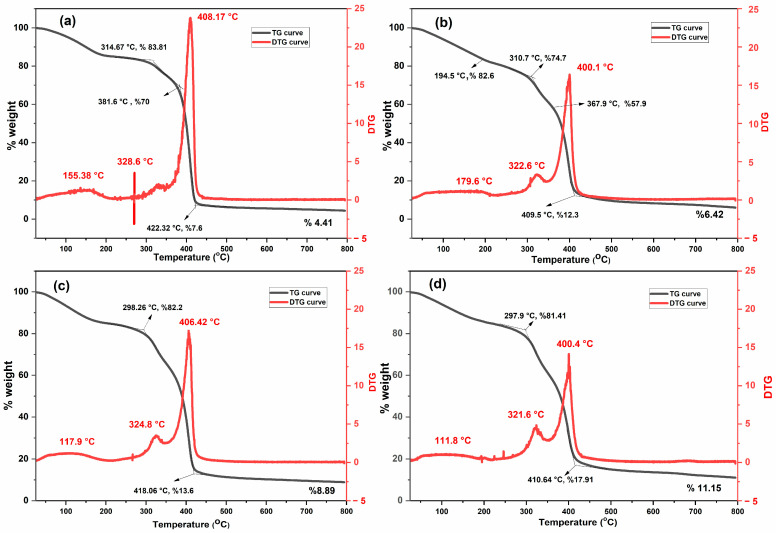
TG and DTG thermograms of (**a**) GEL 1, (**b**) GEL 2, (**c**) GEL 3, and (**d**) GEL 4 hydrogels showing multi-step thermal degradation behavior and composition-dependent thermal stability.

The thermal stability and degradation behavior of MeBSA–PNIPAm hydrogels with varying NIPA:MeBSA feed ratios (85:15, 80:20, 75:25, and 60:40) were investigated by thermogravimetric analysis (TGA) and derivative thermogravimetry (DTG), as shown in Figure 2. It should be noted that increasing MeBSA content is accompanied by a corresponding decrease in the PNIPAm fraction, resulting in a systematic variation of the protein-to-polymer ratio within the hydrogel network.

All formulations exhibited a multistep mass-loss profile, which is characteristic of the hydrogels composed of protein-based segments integrated within a synthetic thermoresponsive polymer backbone [29,41]. At low temperatures, below approximately 120–150 °C, all samples displayed a minor and gradual weight loss. This initial weight loss is attributed to the elimination of physically adsorbed and weakly bound water found in both PNIPAm chains and methacrylated BSA segments. Similar behavior has been widely reported for proteins, polysaccharides, and other natural polymer-based hydrogels [29,41,42,43]. For the temperature range between 200 and 330 °C, a gradual mass decrease was observed for all compositions. This region was accompanied by shoulder-type features in the DTG curves, typically centered around 320–330 °C, and can be attributed to the initial degradation of thermally labile moieties, side-chain scission, and partial cleavage of weaker interactions within the protein–polymer network [29,42,44,45]. The major degradation stage occurred between approximately 250 and 450 °C for all hydrogel compositions. This region corresponds to the thermal scission of the PNIPAm backbone together with the decomposition of protein-derived segments [45]. The DTG curves revealed a single dominant decomposition peak centered around 400–410 °C, indicating that the main mass loss is concentrated within one primary degradation step. The maximum decomposition temperatures were determined to be approximately 408 °C for GEL 1, 400 °C for GEL 2, 406 °C for GEL 3, and 400 °C for GEL 4. These values fall within the characteristic degradation range reported for PNIPAm-based hydrogel systems and confirm that the PNIPAm backbone governs the principal thermal decomposition behavior of the network [29]. Although the main decomposition temperatures of hydrogels are close to each other, clear differences were found in the DTG peak shape and intensity. GEL 1 exhibited the sharpest and most intense DTG peak, which indicates a rapid reduction of mass when compared to other gels. In contrast, increasing MeBSA content resulted in lower and broader DTG peaks, reflecting an increased degradation behavior for protein-rich hydrogels. Similar trends have been reported for PNIPAm-based composite hydrogels including biopolymeric components, where protein or polysaccharide fractions moderate the thermal decomposition profile [42,44,46]. These results indicate that the thermally stable residual mass increased with increasing MeBSA content. At 800 °C, GEL 1 has 4.4% residual mass, whereas GEL 2, GEL 3, and GEL 4 have 6.4%, 8.9%, and 11.1%, respectively. As a result, the total mass loss decreased with increasing protein fraction. This increment in residual mass highlights the contribution of protein-derived domains to the formation of thermally stable residues at elevated temperatures [29,44,47]. Overall, the TGA–DTG results demonstrate that adjusting the NIPA:MeBSA feed ratio effectively modulates the thermal degradation behavior of the hydrogels. While the PNIPAm component is responsible for the primary decomposition stage, the increasing amounts of MeBSA lead to higher mass loss and the enhanced thermal stability of hydrogels. This composition-dependent tunability confirms the hybrid nature of the network and provides a route for tailoring thermal stability in protein–polymer hydrogels intended for biomedical applications.

### 2.3. Phase Transition of Hydrogels (DSC)

**Figure 3:**
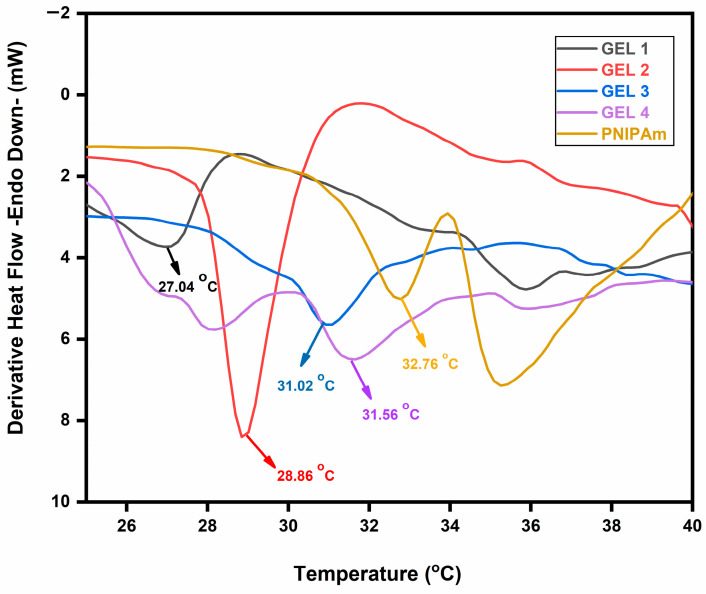
Derivative DSC (endo down) thermograms of fully swollen MeBSA–PNIPAm and pNIPAM hydrogels in water.

The thermoresponsive response of MeBSA–PNIPAm hydrogels was evaluated by Differential Scanning Calorimetry (DSC) using fully swollen MeBSA-PNIPAm hydrogels and pure PNIPAm in deionized water, so that the detected thermal behavior reflects dehydration and rearrangement processes under hydrated conditions relevant to swelling and transport. It should be emphasized that the temperatures determined by DSC correspond to the volume phase transition temperature (VPTT) of chemically crosslinked networks rather than the LCST of linear PNIPAm chains in dilute solution. The derivative DSC thermograms and the corresponding volume phase transition behavior of the hydrogels in water are presented in Figure 3.

In the derivative heat–flow curves (endo down), the endothermic feature is assigned to the dehydration-driven volume phase transition of PNIPAm segments. The pure PNIPAm hydrogel displayed a transition at 32.76 °C, consistent with the characteristic VPTT region of PNIPAm in water [29,44,48]. As shown in Figure 3, incorporation of MeBSA led to a composition-dependent shift in the VPTT of hydrogels. A recent study related to temperature-switchable polymers indicated that transition temperature shifts are governed by changes in the balance of polymer–water and polymer–polymer interactions. From a thermodynamic perspective, such shifts can be interpreted in terms of changes in the Gibbs free energy of mixing (ΔGₘ = ΔHₘ − TΔSₘ), where variations in enthalpic (hydration-related) and entropic (hydrophobic association-related) contributions modulate the apparent transition temperature [27]. Thus, at lower MeBSA content, PNIPAm segment–segment interactions may dominate, facilitating dehydration of polymer chains and resulting in a downward shift of the apparent VPTT. With increasing MeBSA content, the number of hydrophilic and charged amino acid residues in the network increases. These groups increase polymer–water interactions and thus enhance hydration of the hydrogel. Therefore, the entropic balance of the system is modified, and the transition temperature shifts to higher values. Similar effects of various hydrophilic groups and polymer–water interactions on LCST/VPTT have been widely reported for PNIPAm-based systems [27,49].

At lower MeBSA contents (15–20 wt%; GEL 1 and GEL 2), the transition moved significantly to lower temperatures in water, 27.04 °C and 28.88 °C, respectively. This leftward shift suggests an earlier onset of dehydration-mediated structural rearrangement within the hybrid network under water-swollen conditions. Mechanistically, this behavior can be attributed to the amphiphilic and structurally heterogeneous nature of albumin [50,51,52]. The BSA structure contains numerous polar groups and exhibits hydrophobic regions and internal binding sites that may promote hydrophobic interactions with the isopropyl groups of PNIPAm [53,54]. Such interactions probably reduce the effective polymer–water affinity and lower the energetic barrier for dehydration, which is consistent with the fact that stronger hydrophobic association tends to reduce PNIPAm VPTT, whereas increased hydration raises it [50,52]. When the MeBSA content was increased to 25–40 wt% (GEL 3 and GEL 4), the transition temperature shifted back toward that of the pure PNIPAm, reaching approximately 31.56 °C for both formulations (Figure 3). This non-linear behavior may be attributed to the presence of multiple competing effects at higher protein contents. The increased contribution of polar and ionizable amino acid residues probably enhances water retention within the network, while the formation of a more constrained crosslinked structure limits chain mobility. These factors are likely to partially counterbalance the hydrophobic association observed at lower MeBSA contents, resulting in a transition temperature closer to the typical PNIPAm range. In addition, the increased structural heterogeneity may lead to a broader transition, as different regions of the swollen network undergo dehydration over a range of temperatures rather than at a single, well-defined point [47,53]. It should also be noted that DSC measurements were conducted in deionized water to evaluate the thermoresponsive behavior of the crosslinked network without ionic interference. Since swelling and release experiments were performed at pH 1.2 and 7.4, slight shifts in transition behavior are expected under physiological ionic strength and pH conditions. The VPTT of PNIPAm-based networks is known to be sensitive to solvent composition and ionization effects, particularly in hybrid systems containing charged protein domains [49]. Therefore, direct comparison between DSC-derived VPTT values in water and equilibrium swelling behavior in buffer media should be interpreted with caution [54].

### 2.4. Morphology and Macroscopic Appearance of Hydrogels

**Figure 4:**
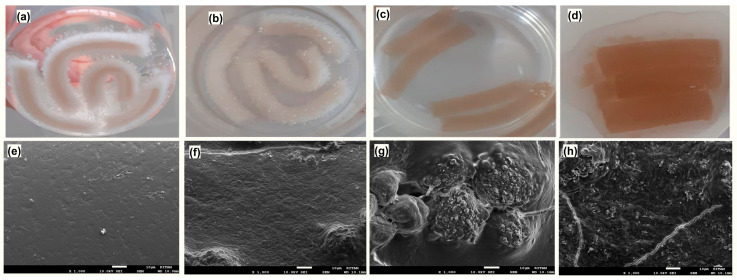
Photographs and SEM images of MeBSA–PNIPAm hydrogels with different compositions. (**a**–**d**) Photographs of swollen hydrogels (GEL 1–GEL 4). (**e**–**h**) SEM images of dried hydrogels (GEL 1–GEL 4, respectively) (1000× magnification; scale bar: 10 μm).

The macroscopic appearance and surface morphology of the MeBSA-PNIPAm hydrogels were examined to evaluate the structural integrity and microstructural characteristics of the gel network. Representative photographs of the swollen hydrogels are presented in Figure 4a–d. All hydrogels preserved their structural integrity after swelling without visible disintegration, indicating successful formation of a stable crosslinked hydrogel network. A noticeable change in color from GEL 1 to GEL 4 was observed, which is consistent with the increasing MeBSA content in the hydrogel formulations.

The surface morphology of the hydrogels was further analyzed using SEM. The dried hydrogels exhibit distinct differences in surface structure depending on composition, as shown in Figure 4e–h. GEL 1 shows a relatively smooth and homogeneous surface, whereas GEL 2 begins to display small surface irregularities. In contrast, GEL 3 and GEL 4 exhibit a more heterogeneous morphology characterized by increased nodular and aggregated surface features. These morphological differences may be attributed to the increased MeBSA, which can promote the formation of protein-rich parts or the aggregation of protein segments within the polymer network, leading to noticeable changes in the surface morphology of the hydrogels [55].

### 2.5. Evaluation of Swelling Behaviors of Hydrogels

**Figure 5:**
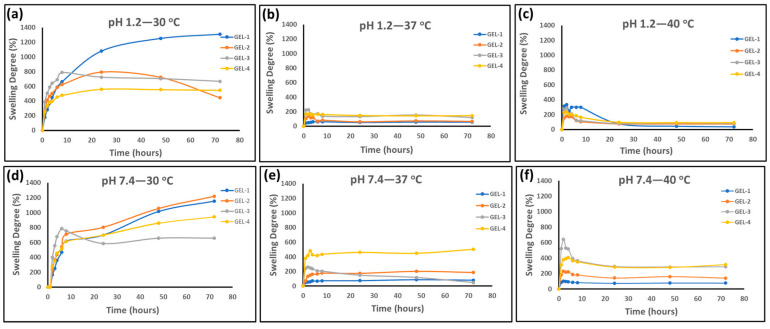
Comparative swelling profiles of MeBSA–PNIPAm hydrogels with different compositions at pH 1.2, 30 °C (**a**); pH 1.2, 37 °C (**b**); pH 1.2, 40 °C (**c**); pH 7.4, 30 °C (**d**); pH 7.4, 37 °C (**e**); pH 7.4, 40 °C (**f**) (*n* = 3 and data are presented as mean).

The swelling experiments of the hydrogels were performed for all compositions (GEL 1 to GEL 4) at two different pH conditions (1.2 and 7.4) and three different temperatures (30, 37, and 40 °C), as presented in Figure 5. The hydrogel compositions were fabricated by increasing the MeBSA content while decreasing the NIPA feed ratio from GEL 1 to GEL 4. The results showed that the swelling behavior of the hydrogels was dependent on both the MeBSA/PNIPAm ratio and environmental conditions. The effect of composition was evaluated to understand how variations in the MeBSA/PNIPAm ratio change the network structure and water uptake capacity. Increasing MeBSA content increases both the number of methacrylate groups participating in covalent network formation [29] and the density of ionizable amino acid groups within the hydrogel matrix [19]. While higher crosslink density restricts network expansion, the increased charged residues enhance electrostatic repulsion under pH conditions far from the isoelectric point. Therefore, the swelling behavior of MeBSA–PNIPAm hydrogels reflects the balance between structural constraints and electrostatic-driven swelling forces.

The selected pH values represent acidic and physiological environments simulating the gastrointestinal route [56], allowing the investigation of the pH-dependent response of the MeBSA component; three different temperatures were chosen to examine the thermoresponsive behavior of the hydrogels [29,31].

The swelling behavior of hydrogels can be evaluated by considering three main contributions: the effective crosslink density arising from methacrylate-mediated covalent network formation, electrostatic repulsion between ionized amino acid residues of MeBSA and the thermoresponsive dehydration of PNIPAm segments above the VPTT. Therefore, the observed swelling trends represent the net result of competing structural and physicochemical interactions within the hybrid network [23,27].

At 30 °C, which is below the characteristic transition temperature of pure PNIPAm (~32 °C) and close to the VPTT region of hybrid hydrogels, all compositions exhibited relatively high swelling at both pH conditions (Figure 5a,d). This behavior reflects that temperature-dependent dehydration of PNIPAm segments is not yet fully dominant within the crosslinked network, allowing the network to retain significant hydration [29,31,54]. It should be noted that swelling experiments were carried out under gastrointestinal-simulating conditions (pH 1.2 and 7.4), whereas DSC measurements were performed in deionized water. Because the VPTT of PNIPAm-based networks depends on solvent composition and pH, slight differences between DSC transition temperatures and swelling results are expected. In addition, the transition in crosslinked hybrid hydrogels occurs over a temperature range rather than at a single sharp point, so partial swelling may still be observed near the VPTT [49,57,58,59].

At pH 1.2, the swelling behavior of the hydrogels exhibited a pronounced temperature dependence. Under strongly acidic conditions (pH 1.2), lysine, arginine, and histidine residues are protonated, resulting in positively charged protein domains. However, the high ionic strength of the acidic medium reduces electrostatic interactions and limits charge repulsion. As a result, PNIPAm dehydration plays a more important role in determining the overall swelling behavior, especially at higher temperatures [23]. At 30 °C, all hydrogel formulations displayed relatively high swelling, consistent with a hydrated and expanded polymer network (Figure 5a). Under these conditions, the swelling followed the order GEL 1 > GEL 2 > GEL 3 > GEL 4. The higher swelling degree observed for GEL 1 can be attributed to its higher PNIPAm content, which promotes water uptake under conditions where network dehydration remains limited rather than fully established [6,60]. GEL 2 showed a less stable swelling profile among other hydrogels, which may be associated with increased network heterogeneity.

Upon increasing the temperature to 37 °C, corresponding to physiological conditions and exceeding the VPTT for all hydrogels, a clear transition in swelling behavior was observed. The swelling order shifted to GEL 4 > GEL 3 > GEL 2 > GEL 1 (Figure 5b). This change indicates temperature-dependent collapse of PNIPAm segments within the crosslinked network becomes the dominant factor controlling swelling, resulting in a reduction in equilibrium swelling for PNIPAm-rich formulations, particularly GEL 1. On the other hand, hydrogels with higher MeBSA content showed relatively higher swelling degrees, suggesting that the hydrophilic and pH-responsive nature of the protein component partially compensates for the network dehydration of PNIPAm domains. Protein-based hydrogels, including albumin-containing systems, are known to exhibit pH-dependent swelling due to changes in protein charge and conformation [58,61,62].

At 40 °C, which is above the VPTT region, the shrinkage of PNIPAm chains became more pronounced (Figure 5c). Although GEL 1 initially exhibited rapid swelling, it subsequently underwent significant deswelling over time. Under these conditions, GEL 4 showed one of the highest swelling degrees, followed by GEL 3 and GEL 2. This behavior can be rationalized by the lower PNIPAm content and higher MeBSA fraction of GEL 4, which increase overall hydrophilicity and promote water uptake when the PNIPAm segments are in a more dehydrated and contracted state within the network [31,58]. Similar reductions in swelling at elevated temperatures have been reported for PNIPAm-based hydrogels, particularly in acidic or high-ionic-strength environments, where polymer–water interactions are weakened [29,57,63]. At pH 7.4, the hydrogels showed high swelling at 30 °C due to the hydrated and expanded state of PNIPAm networks under VPTT (Figure 5d). However, the composition-dependent swelling order differed from pH 1.2 (Figure 5a). The swelling followed the order GEL 2 > GEL 1 > GEL 4, while GEL 3 showed an unusual behavior, displaying high initial swelling followed by a gradual decrease with time. This different behavior between two pH conditions can be attributed to the dominant contribution of the MeBSA component at physiological pH (7.4). Above its isoelectric point (pI 4.7–4.9), bovine serum albumin carries a net negative charge and takes a more expanded and hydrated conformation [19]. This situation enhances hydrophilicity and introduces electrostatic repulsion within the network, which may cause a competition with the swelling behavior of PNIPAm [64,65]. When the pH is far from the isoelectric point, ionization of amino acid residues may increase charge density within the MeBSA domains, potentially generating electrostatic repulsion and contributing to osmotic swelling pressure. The extent of network expansion could therefore depend on whether these electrostatic effects outweigh the constraining influence of crosslink density and PNIPAm-dependent thermoresponsive contraction [23,27].

When the temperature was increased to 37 °C (Figure 5e), exceeding the VPTT region, overall swelling decreased due to thermally induced network dehydration, and the equilibrium swelling order shifted to GEL 4 > GEL 2 > GEL 3 > GEL 1. This trend indicates that MeBSA-rich hydrogels are better able to maintain swelling under physiological conditions, even when PNIPAm dehydration becomes significant. Similar behavior has been reported in PNIPAm-based hybrid and protein-containing hydrogels, where charged or hydrophilic macromolecules partially counteract PNIPAm dehydration and maintain water uptake above the VPTT [4,29,66]. Interestingly, GEL 3 again showed an unusual swelling profile at 37 °C, starting with an initial high swelling followed by a marked decrease over time. This hydrogel appears to have a balanced composition, where crosslink density, charge effects, and PNIPAm contraction are comparable. This balance may explain its distinct swelling behavior over time.

At 40 °C, the swelling order further changed to GEL 3 > GEL 4 > GEL 2 > GEL 1 (Figure 5f). Under these conditions, PNIPAm contraction within the crosslinked network is intensified in NIPA-rich formulations, resulting in very limited swelling for GEL 1, whereas compositions with higher MeBSA content maintain higher swelling.

**Figure 6:**
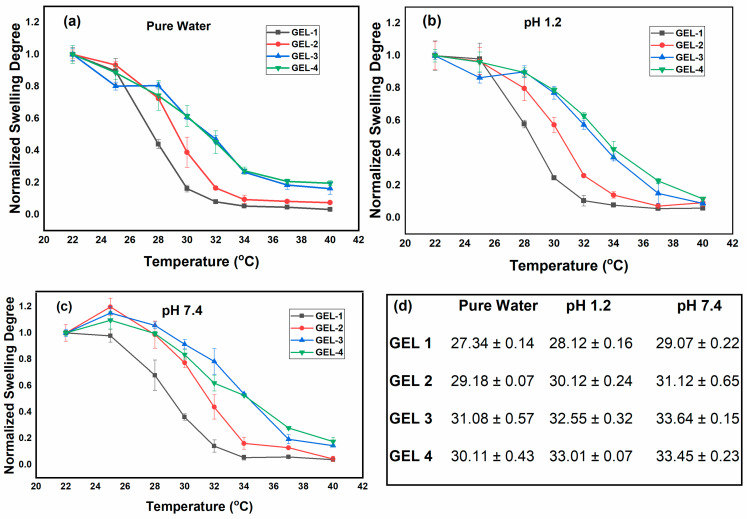
Temperature-dependent normalized swelling behavior and VPTT values of MeBSA–PNIPAm hydrogels under different pH conditions: (**a**) pure water, (**b**) pH 1.2, and (**c**) pH 7.4. (**d**) VPTT values obtained from the inflection points of the curves using Boltzmann fitting. Error bars represent standard deviation (*n* = 3).

Moreover, temperature-dependent swelling tests were carried out to determine the VPTT of the MeBSA–PNIPAm hydrogels in different pH conditions. The swelling degree was measured at various temperatures in distilled water, at pH 1.2 and pH 7.4. The VPTT values were obtained from the inflection points of the swelling–temperature curves using Boltzmann fitting (Figure 6a–c). The VPTT values calculated from these fittings are summarized in Figure 6d. All measurements were performed in triplicate and are reported as mean ± standard deviation, with error bars representing experimental variation.

In pure water, the VPTT values obtained from swelling measurements were 27.43, 29.17, 31.07, and 30.11 °C for GEL 1 to GEL 4, respectively (Figure 6a,d). These values are very close to the transition temperatures obtained from DSC (27.04, 28.86, 31.02, and 31.56 °C), showing good agreement between the two techniques. DSC mainly reflects the thermal dehydration of PNIPAm segments, whereas swelling measurements describe the macroscopic volume change of the hydrogel network.

When the measurements were carried out at different pH values, a clear shift in VPTT was observed. The transition temperatures increased to 28.12–33.01 °C at pH 1.2 (Figure 6b,d) and 29.07–33.64 °C at pH 7.4 (Figure 6c,d), indicating that the pH changes of the environment directly affect the thermoresponsive behavior of the hydrogels. Under acidic conditions (pH 1.2), amino acid groups in BSA become protonated, creating positively charged protein domains. However, the high ionic strength of the acidic medium partially screens electrostatic interactions, so the increase in VPTT compared with water remains limited.

At pH 7.4, which is above the isoelectric point of BSA (pI ≈ 4.7), the protein carries a net negative charge and adopts a more hydrated structure. This higher hydration level and electrostatic repulsion within the network help stabilize the swollen state and shift the thermoresponsive transition to slightly higher temperatures. These observations indicate that the MeBSA–PNIPAm hydrogels respond to both temperature and pH, with the network contraction of PNIPAm chains being influenced by the pH-dependent behavior of the protein component.

Overall, the swelling experiments show that the MeBSA–PNIPAm hydrogels exhibit a tunable and composition-dependent response governed by the combined effects of network structure, PNIPAm thermoresponsive phase transition, and the pH-dependent charge state of the MeBSA component. The observed shifts in VPTT under different pH conditions further confirm that protein ionization and hydration significantly influence the thermoresponsive behavior of the hybrid network. These findings highlight the dual pH- and temperature-responsive nature of the developed hydrogels and support their potential application as stimuli-responsive carriers for controlled drug delivery.

### 2.6. In Vitro Drug Loading and Release Studies of 5-FU

Based on swelling studies, drug loading experiments were performed at 30 °C, to ensure that all hydrogel formulations exhibited their highest swelling degree and thus maximum drug loading capacity [31]. GEL 1 and GEL 2 exhibited high drug loading efficiency (DLE) values of 84 ± 2% and 82 ± 2%, respectively, consistent with their swelling profiles. GEL 3 also showed a relatively high DLE (78 ± 2%), while GEL 4 maintained an unexpected drug loading efficiency (74 ± 2%) despite its lower swelling behavior. Although the loading efficiency gradually decreases with increasing MeBSA content, the relatively high DLE of GEL 4 (74%) suggests that drug–matrix interactions between albumin and 5-FU may partially contribute to drug retention within the hydrogel network [67]. Overall, DLE values above 70% were achieved for each hydrogel, indicating effective drug loading across the entire MeBSA–PNIPAm composition range.

**Figure 7:**
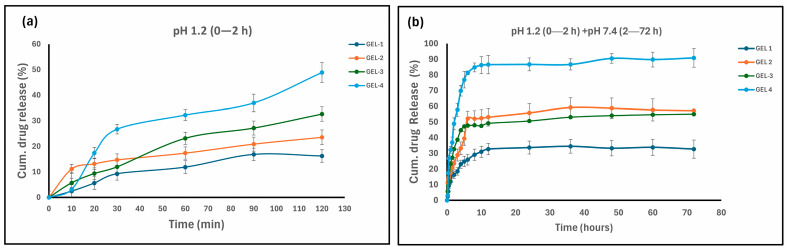
Cumulative 5-FU release profiles of hydrogels with under-simulated gastrointestinal conditions: (**a**) release at pH 1.2 during the initial 0–2 h and (**b**) cumulative release under pH-switch conditions (0–72 h) (*n* = 3 and data are presented as mean ± S.D.).

The cumulative 5-FU release profiles from MeBSA–PNIPAm-based hydrogels were evaluated under simulated gastrointestinal conditions at 37 °C. Drug release was monitored for an initial 2 h at pH 1.2, followed by transfer to pH 7.4 for 70 h. Figure 7a shows the release profiles during the initial 0–2 h (pH 1.2), while Figure 7b presents the cumulative release over 72 h.

These pH-switching conditions were performed to mimic gastric and intestinal environments and to test the stimuli-responsive behavior of the hydrogel network [31,68]. The results show that in vitro 5-FU release for the first 2 h acidic period remained limited for all hydrogels. During this period, GEL 1 exhibited the lowest release (≈15–20%), while GEL 2 and GEL 3 showed moderately higher values (≈25–35%). GEL 4 reached the highest early release (≈45–50%). However, this release remained gradual and did not show an uncontrolled burst. Although there were quantitative differences among the different hydrogel formulations, all hydrogels exhibited partial drug retention under acidic conditions. Following the transfer to 7.4, a pronounced increase in cumulative 5-FU release was observed for all hydrogel formulations (Figure 7b). GEL 1 maintained the lowest overall release, reaching approximately 30–35%, indicating a more restrictive network structure. In contrast, GEL 2 and GEL 3 exhibited moderate and sustained release profiles, with cumulative release values increasing to around 55–60% over the intestinal phase. GEL 4 showed the most pronounced response after the pH transition, with cumulative release rapidly increasing and reaching approximately 90–92%, reflecting a more expanded and responsive hydrogel network under neutral conditions. These differences highlight the strong influence of MeBSA–PNIPAm composition on drug release behavior at pH 7.4 and demonstrate the tunable nature of the hydrogel system. Comparable pH- and stimuli-dependent 5-FU release behaviors have been widely reported in the literature, depending on carrier composition and design [69,70]. For example, Hani et al. reported very high cumulative 5-FU release from colon-targeted cross-linked mastic gum nanoparticles [71], while Tığlı Aydın et al. demonstrated pH-responsive 5-FU release from chitosan-based nanoparticles, with enhanced release at neutral pH [72]. In addition, Luo et al. observed accelerated 5-FU release under acidic conditions in a dual thermo- and pH-sensitive hydrogel system [73]. Beyond these systems, Maghsoudi et al. reported sustained 5-FU release from BSA-based carriers [74], and several PNIPAm-containing hydrogels have been shown to promote enhanced drug release at physiological pH and temperature [31,57,75,76]. Taken together, these reports highlight the diversity of 5-FU release behaviors, while the MeBSA–PNIPAm hydrogels in the present study maintained limited and controlled release during the initial 2 h at pH 1.2. Compared to systems that either strongly suppress gastric release or deliberately promote high acidic release, the present MeBSA–PNIPAm hydrogels display a balanced and tunable release profile. The release at pH 1.2 is limited, while an important increase in cumulative release is achieved after transition to pH 7.4, allowing modulation of drug availability along the gastrointestinal tract. To further elucidate the underlying release mechanisms, the experimental release data were subsequently analyzed using established kinetic models.

### 2.7. Evaluation of Drug Release Kinetics

**Figure 8:**
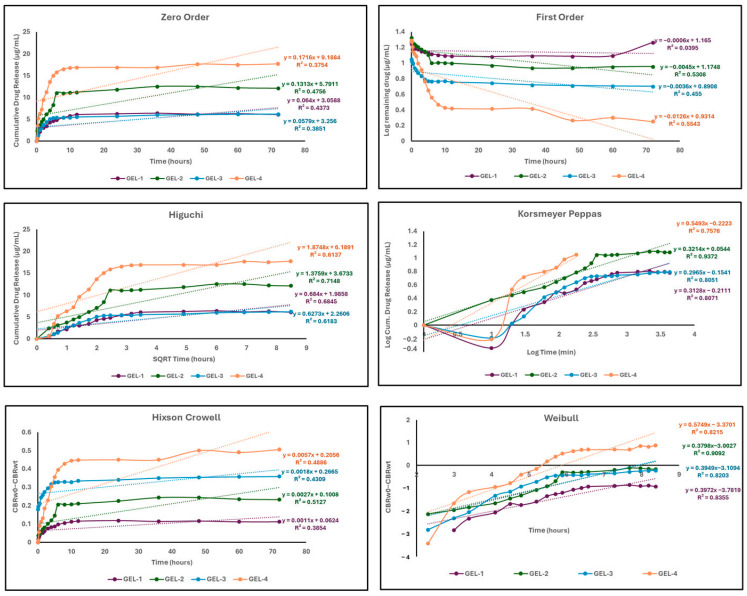
Kinetic models of 5-FU release data from MeBSA–NIPA hydrogels.

The release kinetics of 5-FU from MeBSA–PNIPAm hydrogels were analyzed using different kinetic models, such as zero-order, first-order, Higuchi, Korsmeyer–Peppas, Hixson–Crowell, and Weibull models, in order to better elucidate the release behavior and mechanisms. The kinetic parameters and corresponding R^2^ values are presented in Table 1, and the plots obtained from fitting data to different kinetic models are shown in Figure 8. The correlation coefficients obtained from the fitted plots clearly show that 5-FU release from MeBSA-PNIPAm hydrogels is best described by the Weibull model, which provided the highest R^2^ values for all formulations (0.8203–0.9092). The Korsmeyer–Peppas model also showed a moderate to high fit with the correlations of R^2^ = 0.7576–0.9372, indicating that these two models are the most suitable for describing the release behaviors of the MeBSA–PNIPAm hydrogels. The relatively high correlation values obtained from these models suggests that 5-FU release from the hydrogel networks is mainly governed by a complex transport process rather than by an single-rate mechanism [77].

According to the Korsmeyer–Peppas analysis, the release exponent (*n*) varied between 0.2965 and 0.5493, depending on the hydrogel composition. GEL 1, GEL 2, and GEL 3 exhibited *n* values below 0.45, which is indicative of a Fickian diffusion-controlled release. Accordingly, drug transport is mainly driven by molecular diffusion through the hydrated polymeric network. In contrast, GEL 4 displayed a higher *n* value of 0.5493, indicating a shift toward non-Fickian anomalous transport, likely arising from the increased contribution of polymer chain relaxation associated with the higher MeBSA content [77]. The Weibull model also supports these observations, with the shape parameter β values ranging from 0.3798 to 0.5749, and being under 0.75 for all formulations. These values indicate diffusion-dominated release behavior. Notably, GEL 4 has the higest β value, suggesting a relatively more pronounced release progression at later time points compared to the other hydrogels.

Moreover, the Weibull scale parameter α showed a distinct composition-dependent variation. A considerable decrease in α was observed from GEL 1 to GEL 4, suggesting an acceleration of the release process with increasing MeBSA content. This trend reflects changes in the internal network structure of the hydrogels, which directly influence drug diffusion pathways [78].

The Higuchi model also supports the diffusion-controlled release process [79], which yielded moderate correlation coefficients, between 0.6137 and 0.7148. Although the Higuchi model confirms the contribution of diffusion, it alone is not fully sufficient to explain the release behavior under pH switching conditions.

The zero-order, first-order, and Hixson–Crowell models showed low correlation coefficients for all hydrogels, demonstrating that constant-rate release, purely concentration-dependent kinetics, or erosion-controlled mechanisms do not play a dominant role in the MeBSA–PNIPAm hydrogel systems [79].

Overall, the kinetic analysis of 5-FU release from hydrogels reveals that release is predominantly diffusion-controlled, with clear composition-dependent variations. Changing the ratio of MeBSA/PNIPAm effectively alters the hydrogel network architecture, enabling fine tuning of the drug release rate. The distinct behavior observed for GEL 4 highlights the role of MeBSA content in regulating the release performance of these hydrogels, highlighting their potential for composition-tailored drug delivery applications. These findings are consistent with our previous MeHA–PNIPAm-based hydrogel study, in which 5-FU release was best described by the Weibull and Korsmeyer–Peppas models, confirming a Fickian diffusion-controlled mechanism with composition-dependent tunability [31].

Similar diffusion-controlled release behavior of 5-FU has been widely reported for different hydrogel and nanocarrier systems. For example, chitosan-polyacrylic acid-based Fe_3_O_4_ magnetic nanohydrogels showed Fickian diffusion at both acidic and physiological pH, as confirmed by Higuchi and Korsmeyer–Peppas models [80]. Likewise, hydroxyapatite–gelatin composites followed Higuchi-type kinetics over a broad temperature range, indicating diffusion as the main release mechanism, with temperature and crosslinker content mainly affecting the initial burst [81]. PNIPAm-based injectable pH/thermo dual-sensitive chitosan hydrogels exhibited either diffusion or relaxation assisted release, depending on composition [82], while thermosensitive chitosan hydrogels demonstrated zero-order 5-FU release [83], highlighting the role of polymer matrix control. Overall, these studies show that the formulation ingredients and network structure dominate the release behavior of 5-FU from crosslinked, stimuli-responsive hydrogels.

### 2.8. In Vitro Cytocompatibility Tests of Hydrogels

**Figure 9:**
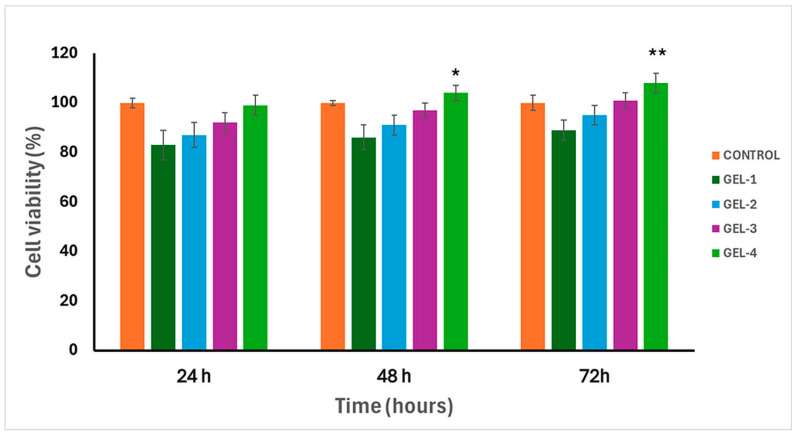
Cell viability of MeBSA-PNIPAm-based hydrogels for 24, 48, and 72 h (*n* = 3 and data are presented as mean ± S.D.). Statistical significance is indicated as * *p* < 0.05 and ** *p* < 0.01 compared to the control group.

The cytocompatibility of the four different hydrogel compositions was evaluated using L929 fibroblast cells after 24, 48, and 72 h of incubation time (Figure 9). All hydrogels showed cell viability values above 80% at all time points, indicating good cytocompatibility. At 24 h, cell viability values of the hydrogels for each composition were slightly lower than those of the control, which may be attributed to initial cell–material interactions. Within prolonged incubation time, a composition-dependent increase in cell viability was observed. In particular, GEL 4 showed significantly higher cell viability compared to the control at 48 h and 72 h, with values slightly exceeding 100%. This increase is commonly observed in MTT assays and is generally associated with enhanced cellular metabolic activity and proliferation rather than any cytotoxic effect. This enhanced cellular response can be associated with the lower PNIPAm content and higher MeBSA content of GEL 4, which likely provides a more hydrated and biologically favorable microenvironment for fibroblast proliferation. Similar trends have been reported for PNIPAm-based hybrid hydrogels containing higher proportions of biopolymers or proteins, where reduced PNIPAm content led to improved fibroblast viability at physiological conditions [42,44]. Overall, these results confirm that all hydrogel formulations are cytocompatible, with GEL 4 exhibiting the most favorable cell response due to its compositional characteristics. Although the cytocompatibility of the hydrogel matrix was confirmed using L929 fibroblast cells, the anticancer activity of the released 5-FU was not directly evaluated on cancer cell lines in the present study. Such biological evaluation would provide further insight into the therapeutic performance of the system and will be addressed in future studies.

## 3. Conclusions

This study successfully synthesized methacrylated bovine serum albumin (MeBSA) with a methacrylation degree of approximately 40%, as confirmed by FTIR and ^1^H-NMR analyses, used together with PNIPAm for the fabrication of dual pH- and temperature-responsive hydrogels for controlled delivery of 5-FU. The methacrylation of BSA enabled the formation of stable covalent crosslinks within the network without the use of additional toxic crosslinkers, providing a structurally stable and biologically favorable protein-based hydrogel. DSC analysis revealed composition-dependent shifts in the volume phase transition temperature, indicating that incorporation of MeBSA modulated the thermoresponsive behavior of the PNIPAm network, while TGA-DTG results demonstrated improved thermal stability in MeBSA-rich compositions. Swelling experiments further demonstrated that the hydrogel hydration and structural response were strongly governed by temperature, pH and the MeBSA/PNIPAm ratio. Hydrogels exhibited high drug loading efficiency (DLE) values of 84 ± 2%, 82 ± 2%,78 ± 2%, 74 ± 2%, respectively, consistent with their swelling profiles, confirming effective drug incorporation. Although hydrogels with higher MeBSA content exhibited reduced swelling capacity, they maintained substantial drug encapsulation efficiency, suggesting that specific interactions between MeBSA and 5-FU may contribute to drug retention within the network. Release studies under pH-switching conditions showed limited and controlled release in acidic medium followed by enhanced cumulative release at pH 7.4, with clear compositional differences among hydrogels. Increasing MeBSA content in GEL 4 significantly increased cumulative release and diffusion rates, highlighting the strong compositional control over network permeability. Among the applied kinetic models, Weibull and Korsmeyer–Peppas models provide the best fits, demonstrating mainly diffusion-controlled 5-FU release. Weibull scale parameters further confirmed composition-dependent modulation of release time, elucidating the tunability of the system. All hydrogels were found to be cytocompatible, while increased MeBSA content enhanced fibroblast viability, underscoring the advantage of protein-based networks for biomedical applications.

Overall, the MeBSA–PNIPAm hydrogels integrate crosslinker-free covalent structuring, dual stimuli responsiveness, tunable diffusion-controlled release, and excellent cytocompatibility with improved thermal stability. The controlled gastric retention, increased intestinal release and protein-based biocompatibility highlight the potential of MeBSA/PNIPAm hydrogels as stimuli-responsive platforms for localized gastrointestinal drug delivery. By adjusting the MeBSA/PNIPAm ratio, release profiles can be precisely tailored, making this protein–polymer hybrid platform a promising candidate for advanced drug delivery systems and next-generation smart biomaterials.

## 4. Materials and Methods

### 4.1. Materials

Bovine serum albumin (BSA, CAS: 9048-46-8), 5-fluorouracil (5-FU, CAS: 51-21-8), phosphate-buffered saline (PBS) tablets, 12–14 kDa cut-off dialysis tubing, and hydrochloric acid (HCl) were purchased from Sigma-Aldrich (St. Louis, MO, USA). N-isopropylacrylamide (NIPA), glycidyl methacrylate (GMA), N,N,N′,N′-tetramethylethylenediamine (TEMED), and potassium peroxydisulfate (KPS) were obtained from Aldrich, Fluka (Buchs, Switzerland), Analyticals Carlo-Erba (Milan, Italy), and J.T. Baker (Phillipsburg, NJ, USA), respectively. All chemicals were used as received without further purification.

### 4.2. Equipment

Thermogravimetric analysis (TGA) was performed using a TA Instruments SDT-Q600 simultaneous TGA/DSC analyzer (TA Instruments, New Castle, DE, USA) to evaluate the thermal stability and decomposition behavior of the dried hydrogels. Measurements were carried out in the temperature range of 25–800 °C at a heating rate of 10 °C/min under a nitrogen atmosphere, with a gas flow rate of 50 mL/min and using alumina pans.

The volume phase transition behavior of fully swollen MeBSA–PNIPAm hydrogels was investigated by differential scanning calorimetry (DSC) using a PerkinElmer Jade-type DSC (PerkinElmer, Waltham, MA, USA) under an argon atmosphere at a heating rate of 10 °C/min. The derivative DSC (DDSC) curves were obtained using Pyris TA software (version 7). Prior to the measurements, the DSC instrument was calibrated using indium and zinc standards.

Fourier transform infrared (FT-IR) spectra of BSA, GMA, and methacrylated BSA (MeBSA) were recorded using a Thermo Nicolet 6700 FT-IR spectrophotometer (Thermo Fisher Scientific, Waltham, MA, USA) equipped with a diamond attenuated total reflectance (ATR) accessory (Smart Orbit). Spectra were collected in the range of 400–4000 cm^−1^.

^1^H Nuclear Magnetic Resonance (^1^H-NMR) characterization was performed using a Varian 600 MHz NMR spectrometer (Agilent Technologies, Santa Clara, CA, USA) in D_2_O at 37 °C.

The surface morphology of the hydrogels was examined using scanning electron microscopy (SEM) (JEOL Ltd., Tokyo, Japan). Prior to imaging, the hydrogel samples were dried at room temperature and mounted on aluminum stubs using conductive carbon tape. SEM images were obtained using a JEOL JSM-7001F field emission scanning electron microscope (JEOL Ltd., Tokyo, Japan) operated at an accelerating voltage of 10 kV.

Drug release experiments were carried out using an N-Biotek NB-205 shaking incubator (N-Biotek Inc., Bucheon, Republic of Korea) operated at 37 °C and 130 rpm. The release of 5-fluorouracil (5-FU) was monitored in buffer solutions at pH 1.2 and pH 7.4 using a PerkinElmer Lambda-35 UV–Vis spectrophotometer (PerkinElmer, Waltham, MA, USA) over a wavelength range of 200–400 nm. The percentage of drug released was calculated using calibration curves prepared separately in pH 1.2 and pH 7.4 buffer solutions.

### 4.3. Synthesis of Methacrylated BSA

The methacrylation procedure of BSA was adapted from a previously reported method in the literature [20], with minor modifications. Briefly, BSA (4 g) was dissolved in 20 mL of PBS, followed by the dropwise addition of 500 μL of glycidyl methacrylate (GMA) under an argon atmosphere with continuous stirring. The reaction proceeded for 7 days at room temperature. Subsequently, the mixture underwent 5 days of dialysis against deionized water using a 14 kDa molecular weight cut-off membrane, with periodic medium replacement. The resulting solution was then lyophilized to yield the desired product. The conversion of BSA was determined from ^1^H-NMR spectroscopy as % 40.

### 4.4. Preparation of MeBSA-PNIPAm Hydrogels

MeBSA–PNIPAm hydrogels were prepared using four different initial feed ratios of NIPA monomer to MeBSA (85:15, 80:20, 75:25, and 60:40 wt%) by adapting a redox polymerization method reported in our previous work [29,31]. The initial NIPA monomer/MeBSA feed ratios are summarized in Table 2

**Figure 10:**
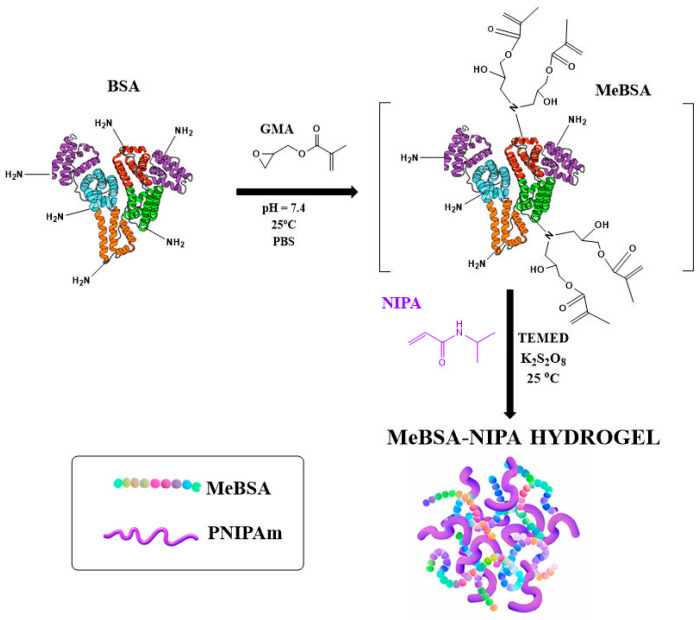
Schematic representation of MeBSA–PNIPAm hydrogel preparation method.

For the first formulation, 0.1 g of MeBSA was dissolved in 10 mL of ultrapure water in a 20 mL test tube. Then, 0.425 g of NIPA and 0.2 mL potassium peroxydisulphate solution (0.05 M) were added. The mixture was purged with argon for 15 min to remove dissolved oxygen. Afterwards, 0.2 mL TEMED solution (0.05 M) was added, and the solution was purged again with argon for an additional 2 min. Afterwards, the reaction mixture was poured into a plastic straw (5 cm long, 5 mm diameter) and left at room temperature for crosslinking. The hydrogels were purified by three swelling–deswelling cycles at 20 °C and 40 °C to eliminate unreacted materials and afterwards dried at 40 °C under vacuum for 1 week. The schematic representation of the hydrogel preparation is presented in Figure 10.

### 4.5. Determination of Swelling Behavior of Hydrogels

Swelling experiments were performed for MeBSA–PNIPAm-based hydrogels with four different compositions under two pH conditions, pH 1.2 and pH 7.4, to simulate the gastrointestinal environment. The acidic medium (pH 1.2) was prepared by dissolving 2.0 g of sodium chloride in distilled water, adding 7.0 mL of hydrochloric acid, and diluting the solution to 1 L. The pH was then carefully adjusted to 1.2 using hydrochloric acid. The pH 7.4 buffer was prepared using PBS tablets, and the pH was checked with a calibrated pH meter. In addition, three different temperatures (30, 37, and 40 °C) were selected to evaluate the thermoresponsive behavior of the hydrogels, corresponding to temperatures below, near, and above the VPTT of PNIPAm-based systems. For each experiment, dried hydrogel samples were first weighed (*W*_0_) and immersed in the swelling medium of the desired pH and selected temperature. The samples were removed from the medium at predefined time intervals, gently blotted with filter paper and reweighted (*Wₜ*). The samples were then returned immediately to the same medium to continue the swelling process, until no further change in sample mass was observed. The constant mass indicated that equilibrium swelling had been reached [31,84]. The percent swelling degree (*SD*, %) was calculated gravimetrically using Equation (2):

(2) $$\%\;SD=\;\frac{W_{s}-W_{d}}{W_{d}}\;\times 100$$

where *W_d_* is the dry mass and *W_s_* is the swollen mass of hydrogels.

Additionally, to determine the VPTT, temperature-dependent swelling measurements (22–40 °C) were performed in deionized water for pH 1.2 (HCl) and pH 7.4 (PBS) buffers. The equilibrium swelling degree was evaluated as a function of temperature. For comparison of the temperature- and pH-dependent behavior, swelling values were normalized with respect to the equilibrium swelling degree measured at 22 °C. The normalized swelling curves were analyzed using Origin software and fitted with the Boltzmann equation. The VPTT values of hydrogels were determined from the inflection points of the fitted curves. All measurements were carried out in triplicate, and the results are reported as mean ± standard deviation.

### 4.6. In Vitro Loading and Release Studies of 5-FU

Individual dried hydrogels were weighed and immersed in separate 40 mL solutions of 5-FU (2.5 mg/mL) at 30 °C for three days to allow equilibrium swelling and maximum drug loading. After loading, the hydrogels were removed from the solution, rinsed with distilled water to remove excess drug from the surface, gently blotted with filter paper, and weighed again. The amount of absorbed 5-FU was calculated as weight percent based on the dry weight of the hydrogels. All loading experiments were performed in triplicate, and the results are reported as mean values. The release studies were performed under simulated gastrointestinal conditions. The 5-FU-loaded hydrogels were placed in 40 mL of buffer solution at pH 1.2 and pH 7.4. The samples were incubated at 37 °C, with shaking at 120 rpm. At predetermined time intervals, 3 mL of the release medium was withdrawn and replaced with the same volume of fresh buffer at the corresponding pH to keep the total volume constant. The amount of released 5-FU was measured by UV–Vis spectroscopy at 265 nm and converted to concentrations using calibration curves prepared separately for pH 1.2 and pH 7.4. Drug loading efficiency and cumulative release were calculated using Equations (3) and (4), respectively [85], and all values are given as averages of three independent experiments.

(3) $$Cumulative\;Drug\;Release=\left[C_{n}+\frac{3}{40}\sum C_{n-1}\right]\times 1000$$

(4) $$Drug\;Loading\;Efficiency=\frac{5-FU\;weight\;in\;hydrogel}{dry\;hydrogel\;weight}$$

where *C_n_* and *C_n_*_−1_ denote the amounts of drug released at the current (*n*) and the previous (*n* − 1) sampling times, respectively.

### 4.7. Kinetic Investigation of 5-FU Release

To investigate the kinetic release mechanism of 5-FU from MeBSA–NIPA-based hydrogels, the in vitro release data obtained under simulated gastrointestinal conditions (pH 1.2 and 7.4) were fitted to several conventional kinetic models. The corresponding model equations are summarized in Table 3.

Within the applied kinetic models, $M_{t}$ denotes the amount of drug released at a given time *t*, whereas $M_{0}$ refers to the initial drug content and $M_{\infty }$ represents the total amount of drug released at infinite time. The fractional release, F(t), is expressed as the ratio of $M_{t}$ to $M_{\infty }$. The constants $k_{0}$, $k_{1}$, $k_{H}$, k, and $k_{H-C}$ correspond to the release rate parameters derived from the zero-order, first-order, Higuchi, Korsmeyer–Peppas, and Hixson–Crowell models, respectively. In addition, the release exponent n in the Korsmeyer–Peppas model characterizes the time-dependent release behavior, while the Weibull parameters α and β are associated with the characteristic release time (63.2% of total release) and the overall shape of the release profile [31,85,86,87].

### 4.8. In Vitro Cytocompatibility Tests of Hydrogels

The cytocompatibility of the hydrogel formulations was evaluated using L929 fibroblast cells (ATCC CCL-1, American Type Culture Collection, Manassas, VA, USA). Cells were cultured in Dulbecco’s Modified Eagle Medium (DMEM) supplemented with 10% fetal bovine serum and 1% penicillin–streptomycin under standard conditions (37 °C, 5% CO_2_). Hydrogel samples were sterilized by UV irradiation and equilibrated in phosphate-buffered saline. Cells were seeded in 24-well plates and incubated with the hydrogel samples, while cells cultured without hydrogels served as the control group. Cell viability was assessed after 24, 48, and 72 h using a colorimetric assay, and absorbance was measured with a microplate reader. Cell viability was calculated as a percentage relative to the control group. All experiments were performed in triplicate (*n* = 3), and results are presented as mean ± standard deviation (SD). Statistical analysis was conducted using one-way ANOVA followed by Tukey’s test, with *p* < 0.05 considered statistically significant.

### 4.9. Statistical Analysis

All results were expressed as the means of three separate experiments (*n* = 3), and data were displayed as mean ± SD. Data from the experiments were compared using one-way ANOVA followed by Tukey’s test, with *p* < 0.05 considered significant (Microsoft Excel for Office 365 (Microsoft Corporation, Redmond, WA, USA)). The temperature-dependent swelling data used for VPTT determination were analyzed using Origin software 2018 (OriginLab Corporation, Northampton, MA, USA), and error bars in the graphs represent standard deviation.

## Figures and Tables

**Table 1:** Kinetic parameters and correlation coefficients (R^2^) obtained from fitting data to different kinetic models.

Models	Parameters	GEL 1	GEL 2	GEL 3	GEL 4
Zero-Order	R^2^	0.4373	0.4756	0.3851	0.3754
	k_o_	0.064	0.1313	0.0579	0.1716
First-Order	R^2^	0.0395	0.5308	0.455	0.5543
	k_1_	0.00138	0.01036	0.00829	0.02902
Higuchi	R^2^	0.6845	0.7148	0.6183	0.6137
	k_h_	0.684	1.3759	0.6273	1.8748
Korsmeyer–Peppas	R^2^	0.8071	0.9372	0.8051	0.7576
	n	0.3128	0.3214	0.2965	0.5493
	kk-p	0.61504	1.13344	0.71598	0.59938
Hixson–Crowell	R^2^	0.3854	0.5127	0.4309	0.4886
	k_H-C_	0.0011	0.0027	0.0018	0.0057
Weibull	R^2^	0.8355	0.9092	0.8203	0.8215
	α	13650	2715	2628	352
	β	0.3972	0.3798	0.3949	0.5749

**Table 2:** Initial feed composition of hydrogel formulations.

Hydrogel Code	NIPA Monomer (g)	MeBSA (mg)	NIPA in Feed (wt%)	MeBSA in Feed (wt%)
GEL 1	0.425 g	0.075 g	85	15
GEL 2	0.400 g	0.100 g	80	20
GEL 3	0.375 g	0.125 g	75	25
GEL 4	0.300 g	0.200 g	60	40

**Table 3:** Kinetic models and corresponding equations used for the analysis of 5-FU release.

Model	Equation
Zero-order	$M_{t}=M_{0}+k_{o}.t$
First-order	$\ln(M_{0}-M_{t})=\ln M_{0}-k_{1}.t$
Higuchi	$M_{t}={\;k}_{H}.t^{1/2}$
Korsmeyer–Peppas	$\frac{M_{t}}{M_{\infty }}=k.t^{n}$
Hixson–Crowell	$M_{0}^{\frac{1}{3}\;}-M_{t\;}^{\frac{1}{3}\;}=k_{H-C}.t$
Weibull	$F\left(t\right)=1-exp\left[-(\frac{t}{\alpha }{)}^{\beta }\right]$

## Data Availability

The raw data supporting the conclusions of this article will be made available by the authors on request.

## Abbreviations

The following abbreviations are used in this manuscript:

ANOVA	Analysis of Variance
ATR	Attenuated Total Reflectance
BSA	Bovine Serum Albumin
CO_2_	Carbon Dioxide
DDSC	Derivative Differential Scanning Calorimetry
DLE	Drug Loading Efficiency
DMEM	Dulbecco’s Modified Eagle Medium
DSC	Differential Scanning Calorimetry
DTG	Derivative Thermogravimetry
D_2_O	Deuterium Oxide
FTIR	Fourier Transform Infrared Spectroscopy
GMA	Glycidyl Methacrylate
HCl	Hydrochloric Acid
LCST	Lower Critical Solution Temperature
MeBSA	Methacrylated Bovine Serum Albumin
NIPA	N-Isopropylacrylamide
NMR	Nuclear Magnetic Resonance
PBS	Phosphate-Buffered Saline
PNIPAm	Poly(N-isopropylacrylamide)
SD	Standard Deviation
SD (%)	Swelling Degree
SEM	Scanning Electron Microscopy
TEMED	N,N,N′,N′-Tetramethylethylenediamine
TGA	Thermogravimetric Analysis
UV	Ultraviolet
UV–Vis	Ultraviolet–Visible Spectroscopy
VPTT	Volume Phase Transition Temperature
5-FU	5-Fluorouracil
